# Effects of different addition levels of CHM-JM113 on growth performance, antioxidant capacity, organ index, and intestinal health of AA broilers

**DOI:** 10.3389/fvets.2024.1388173

**Published:** 2024-05-15

**Authors:** Guanhua Fu, Mengyu Zhang, Yuanyuan Huang, Runyu Han, Kaixuan Qi, Lidong Yin, Dongchen Zhao, Yueyan Huang, Tenghe Ma, Lihong Wang

**Affiliations:** ^1^College of Life Science and Food Engineering, Hebei University of Engineering, Handan, China; ^2^Breeding Branch, Muyuan Foods Co., Ltd., Nanyang, China

**Keywords:** Chinese herbal medicine, JM113, growth performance, antioxidant capacity, organ index, intestinal health, AA broiler

## Abstract

The purpose of the present study was to investigate the effects of different levels of a Chinese herbal medicine formulation combined with JM113 (CHM-JM113) on growth performance, antioxidant capacity, organ index, and intestinal health of AA broilers. The AA broiler chicks were randomly allocated to 5 treatments as follows: a basic diet for the control group, the basic diet supplemented with 0.25% CHM-JM113, 0.5% CHM-JM113, 1% CHM-JM113 and 2% CHM-JM113 for the treatment group, respectively. The results showed that the addition of CHM-JM113 to the diet significantly reduced the mortality (*p* < 0.01) and improved the European Broiler Index (EBI) (*p* < 0.05), whereas it had no significance on growth performance of AA broilers (*p* > 0.05). Comparing the control group, 0.5 and 1% CHM-JM113 group significantly improved the organ index of liver, spleen and bursa (*p* < 0.05). In terms of intestinal morphology and structure, the addition of different levels of CHM-JM113 increased VH and VH/CD ratio, decreased CD in the small intestine compared to the control group, with 1 and 2% of the additive dose being more effective (*p* < 0.05). Chinese herbal medicine and probiotics as natural antioxidants also significantly increased the content of SOD in serum of 21-day-old broilers (*p* < 0.01), and significantly decreased the content of MDA in serum (*p* < 0.01). At 42 days of age, the addition of 1 and 2% CHM-JM113 significantly increased the content of SOD (*p* < 0.01) and significantly decreased the content of MDA in the organism (*p* < 0.01), accompanied by a significant increase in T-AOC and CAT content. In the study of the effect of CHM-JM113 on intestinal immunity, compared with the control group, we found that 1% or 2% CHM-JM113 had a better effect on the expression of occludin and claudin-1 in the intestinal segments of broilers (*p* < 0.05). For the expression of GATA-3, 0.5% CHM-JM113 may have a better effect (*p* < 0.05). CHM-JM113 may be used as an antibiotic alternative in broiler production.

## 1 Introduction

Antibiotics paly an essential role in animal husbandry and are generally used to prevent, treating diseases and enhancing growth (1, 2). However, the extensive use of antibiotics will lead to drug resistance of the harmful strains (3, 4), negatively impact the quality of livestock products (5, 6), and even destroy the ecological environment (7–9), endangering human health (5, 10–12). For agriculture, there is an urgent need to find alternatives to antibiotics in edible animals, especially poultry and livestock (1, 13). Probiotics, prebiotics, synbiotics, organic acids, essential oils, enzymes, immunostimulants, and plant-based (plant-derived) substances, including herbs, botanicals, essential oils, and oleoresins, are the most common feed additives for substituting antibiotics (13, 14).

Chinese herbal medicine and its extracts are natural and effective, with few side effects and the risk of inducing bacterial resistance is minimal (15), and have gradually become the preferred alternative to antibiotics (16). Chinese herbal medicine contains a variety of active ingredients, such as plant polysaccharides, chlorogenic acid, alkaloids, essential oils, flavonoids, plant polyphenols, sterols and so on. These active substances enhance the antioxidant capacity and immunity of animal bodies and limit the formation of dangerous bacteria (17–19). Thyme, Astragalus, tangerine peel and dandelion are traditional Chinese medicinal materials, which play a certain role in bacteriostasis, promoting animal growth and development, and enhancing animal immunity. The addition of thyme could improve the activity of SOD and GSH-Px in the blood of rats, reduce the level of malondialdehyde and boost the antioxidant capacity of rats (20). Astragalus polysaccharide in Astragalus has the advantages of minimal residue and no tolerance, which increases animal immunity and stimulates animal growth (21). Flavonoids (active ingredients in tangerine peel) contain phenolic hydroxyl structure, which could be combined with free radicals to form complex free radicals, reduce the production of free radicals and reduce the damage of free radicals to the body, so as to achieve the effect of anti-oxidation (22, 23). Adding 500 mg/kg dandelion tannins increased the ADG of Wenchang chickens, stimulated intestinal development, and increased the absorption rate of nutrients in the small intestine (24). It also enhance the daily feed intake (ADFI) of AA broilers, reduced the feed/gain ratio (F/G), and promoted the expression of claudin and occludin-1 in the intestine (25). The effects of Chinese herbal medicine mixtures on animal body vary with its unique circumstances. Herbal mixtures composed of numerous herbs contain a variety of active ingredients, which may demonstrate higher biological efficacy than single herb extracts (26).

In addition, probiotics also play a crucial role in resistance. Probiotics colonize in the digestive tract of animals, minimize the harm of pathogenic bacteria to the intestinal tract, protect the integrity of the intestinal structure, and stimulate the intestinal development of the animals (27). *Lactobacillus plantarum* JM113 has high antioxidant activity, which could enhance the digestion, absorption and metabolism of intestinal tract under DON pollution by reducing the damage of intestinal morphology and intestinal barrier, increasing the abundance of beneficial bacteria and regulating the balance of intestinal flora (28). Li et al. (29) found that the combination of non-antibiotic growth promoters (NAGPCs) had superior effect than the usage of non-antibiotic growth promoter alone. However, the combination of the same type of NAGPC may limit the beneficial benefits of synergy.

Based on the above background, it is considered whether the combined use of Chinese herbal medicine and probiotics may have a cumulative effect on the alternative use of antibiotics. This study was conducted to study the effects of Chinese herbal medicine compound preparation and probiotics preparation on growth performance, antioxidant capacity, organ index and intestinal health of broilers, and to provide potential alternatives to antibiotics for use in broiler production.

## 2 Materials and methods

### 2.1 Parameters experimental design, characteristics of objects and conditions of research

A total of 450 healthy 1-day-old AA broilers with similar body weight (40.0 ± 5.0 g) were randomly divided into 5 groups, with 6 replicates per group and 15 broilers per replicate. The animal protocols used in this study have been evaluated and approved by the Institutional Animal Care and Use Committee of Hebei University of Engineering (Identification code: HBUE2022, Date of approval: 16 March 2022). The control group was provided a basic diet, while the experimental groups were fed the basic diet supplemented with 0.25, 0.5, 1 and 2% CHM-JM113 (a Chinese herbal medicine formulation combined with JM113), respectively. During the feeding phase, all experimental groups were supplemented with JM113 powder (1 g/kg in the meal). The experiment lasted for 42 days, which was divided into an early stage (1–21 days old) and a late stage (22–42 days old). The basic diet composition was developed and granulated according to the Chinese broiler nutrition standard (NY/T 33-2004). The food composition and nutritional composition are shown in Table 1. During a 42 day test period, animals were fed in Gaobai Family Farm in Linzhang County, Handan City. Chinese herbal medicine was composed of Astragalus, tangerine peel, dandelion and thyme (brought from Anhui Bozhou Wuhua Food Co., Ltd.) in a ratio of 1:1:1:1. The viable count of *Lactobacillus plantarum* JM113 was 4.4 × 10^9^ CFU/g.

**Table 1:** Composition and nutrient level of the basal diets (%, air-fed basis).

Items	Content		
	Days 1–21	Days 22–30	Days 31–42
**Ingredients (%)**			
Corn	54.80	55.89	56.98
Soybean oil	3.68	3.83	3.98
Soybean meal	38.45	36.93	35.40
L-lysine HCl	0.20	0.55	0.90
DL-methionine	0.25	0.25	0.24
Calcium hydrogen phosphate	1.92	1.86	1.80
Salt	0.50	0.50	0.50
Vitamin premix^a^	0.10	0.10	0.10
Mineral premix^b^	0.10	0.10	0.10
Total	100.00	100.00	100.00
**Calculation of nutrients** ^c^			
Metabolizable energy, kcal/kg	12.35	12.50	12.64
Crude protein, %	23.00	20.00	18.50
Crude fat, %	5.00	5.00	5.00
Total calcium, %	0.90	0.85	0.80
Available phosphorus, %	0.45	0.42	0.40
Linoleic acid, %	1.00	1.00	1.00
Lysine, %	1.20	1.01	0.94
DL-methionine, %	0.47	0.44	0.38
Threonine, %	0.78	0.76	0.70
Tryptophan, %	0.22	0.19	0.18

a Supplied per kilogram of diet: vitamin A, 1,500 IU; vitamin D3, 750 IU; vitamin B1, 0.8 mg; vitamin B2, 1.6 mg; vitamin B6, 1 mg.

b Supplied per kilogram of diet: copper, 10 mg; manganese, 55 mg; zinc, 65 mg; selenium, 150 mg; potassium, 100 mg; sodium, 100 mg; phytase, 5,000 U.

c The nutrient levels were as fed basis.

During the experiment, free diet, drinking water and artificial light were supplemented. The feeding method was imprisoned in the house. The illumination time was 24 h per day from 1 to 7 days of age, and subsequently the illumination was reduced by 1 h per week. After 4 weeks of age, the illumination time was switched to a standard illumination time of 20 h. The room temperature was 33°C on the first day of the experiment, and then decreased by approximately 2.5°C every week until it reached 19°C, while maintaining a humidity level of 60–70%.

### 2.2 Sample collection

On the 21st and 42nd days of the experiment, one AA broiler was randomly selected from each replicate to collect wing vein serum, which was then stored at −20°C for further use. After bloodletting the broiler’s neck, the neck and abdominal cavity were quickly opened, and the cardiac, liver, spleen, bursa of Fabricius, and other organs were completely removed. The fascia and adipose tissue adhered to the surface of the organs were removed, and the blood on the surface was sucked with filter paper. Accurately weigh and calculate organ index.

### 2.3 Intestinal morphology

The duodenum, jejunum and ileum were excised approximately 2 cm and preserved in 4% paraformaldehyde. The intestinal tissue was dehydrated with ethanol, washed with xylene, soaked in wax, embedded, sectioned (μm), and stained with hematoxylin-eosin (H&E) for morphological measurement. The villus height (VH; measured from the villus-crypt junction to the villus tip) and the crypt depth (CD; measured from the crypt base to the villus-crypt junction) were estimated by the Image-Pro Plus 6.0 software, and the ratio of villus height to crypt depth (VH/CD) was calculated.

### 2.4 Serum antioxidant capacity

According to the manufacturer’s plan (Suzhou Grace Biotechnology Co., Ltd.), superoxide dismutase (SOD) detection kit (G0101W), catalase (CAT) detection kit (G0105W), total antioxidant capacity (T-AOC) detection kit (G0142W) and malondialdehyde (MDA) detection kit (G0109W) were used to determine the levels of SOD (U/mL), CAT (U/mL), T-AOC (U/mL) and MDA (nmol/mL) in serum of AA broilers.

### 2.5 The level of claudin-1, occludin and GATA-3 gene expression in intestinal mucosa

Primers were designed according to the gene sequences of GADPH, claudin-1, occludin and GATA-3 from the GenBank database. Primers for quantitative reverse transcription polymerase chain reaction (qRT-PCR) were designed using Primer 5.0 and the National Center for Biotechnology Information (NCBI). Primer-related information is shown in Table 2. Total RNA was extracted from each segment of the small intestine, and the integrity of the RNA was confirmed through agarose gel electrophoresis. The entire RNA was selected for reverse transcription, and the expression levels of claudin-1, occludin and GATA-3 genes in the intestinal segments of AA broilers were detected by RT-PCR. The expression of the target gene relative to the internal reference gene was calculated by 2^−ΔΔCt^ method.

**Table 2:** Sequences of the primers used for the determination of gene expression levels.

Genes	GenBank No.	Primer Sequence (5′ → 3′)	Length/bp
GAPDH	NM_204305.2	F: GGTGGCCATCAATGATCCCT	105
		R: CCGTTCTCAGCCTTGACAGT	
Claudin-1	NM_001013611.2	F: AGATCCAGTGCAAGGTGTACG	107
		R: AAACACACCAACCAGACCCA	
occludin	NM_205128.1	F: TGAATGCACCCACTGAGTGTT	99
		R: CCAGAGGTGTGGGCCTTAC	
GATA-3	NM_001008444.2	F: CACTTTATCCGCCGGTCACT	179
		R: ACGACTCCAGCTTCATGGTG	

F, forward primer; R, reverse primer.

### 2.6 Statistical analysis

Data were analyzed using SPSS 20.0 (SPSS, Inc., Chicago, Illinois, United States) and compared using one-way analysis of variance (ANOVA) and Duncan’s multiple comparisons. The data is expressed as mean ± standard deviation (SD). *p* < 0.05 was considered significant.

## 3 Results

If there is no specific explanation, the following results were analyzed in comparison with the control group.

### 3.1 Growth performance

As shown in Table 3, dietary CHM-JM113 supplementation had no statistically significant impact (*p* > 0.05) on the ADG, ADFI and F/G during days 1 to 21, days 22 to 42, and during the whole period (days 1 to 42). Dietary supplementation with 0.25, 0.5, 1 and 2% CHM-JM113 significantly reduced (*p* < 0.01) mortality from days 1 to 42, and linearly (*p* < 0.01) and quadratically (*p* < 0.01) decreased the mortality. Additionally, dietary supplementation with 0.5, 1 and 2% CHM-JM113 increased the EBI (*p* < 0.05) for AA broiler breeding from days 1 to 42.

**Table 3:** Effect of experimental treatments on growth performances of broilers during 1 to 42 days of age.

Items	Control	Dietary CHM-JM113 level (%)				SEM	*p*-value		
		0.25	0.5	1	2		ANOVA	Linear	Quadratic
BW, g	45.60 ± 1.61	45.50 ± 1.44	45.60 ± 1.57	45.60 ± 1.30	45.50 ± 1.40	0.321	1.000	0.971	0.975
**Days 1–21**									
ADG, g	44.96 ± 0.43	45.94 ± 3.08	45.58 ± 0.36	45.94 ± 3.64	45.89 ± 2.62	0.543	0.984	0.685	0.803
ADFI, g	59.88 ± 0.30	61.16 ± 5.12	59.00 ± 1.01	60.08 ± 4.01	60.68 ± 3.17	0.740	0.939	0.933	0.796
F/G, g/g	1.33 ± 0.02	1.34 ± 0.16	1.29 ± 0.01	1.31 ± 0.02	1.32 ± 0.01	0.017	0.951	0.734	0.651
**Days 22–42**									
ADG, g	67.08 ± 4.43	71.95 ± 1.50	73.83 ± 2.49	71.24 ± 2.12	71.71 ± 2.92	0.863	0.136	0.134	0.058
ADFI, g	129.64 ± 3.51	130.58 ± 1.50	127.47 ± 4.71	127.21 ± 5.86	129.14 ± 1.73	0.908	0.790	0.547	0.575
F/G, g/g	1.16 ± 0.07	1.11 ± 0.03	1.07 ± 0.02	1.09 ± 0.07	1.08 ± 0.01	0.013	0.174	0.049	0.161
**Days 1–42**									
ADG, g	56.58 ± 2.84	57.27 ± 2.18	57.17 ± 3.39	57.32 ± 1.66	57.21 ± 2.72	0.577	0.996	0.789	0.816
ADFI, g	94.76 ± 1.83	95.87 ± 3.19	93.23 ± 2.70	93.65 ± 0.93	94.91 ± 1.83	0.549	0.629	0.646	0.504
F/G, g/g	1.69 ± 0.07	1.64 ± 0.04	1.65 ± 0.09	1.59 ± 0.05	1.67 ± 0.05	0.016	0.463	0.521	0.220
Mortality, %	19.30 ± 3.04^a^	15.79 ± 2.67^b^	1.75 ± 3.04^d^	2.08 ± 3.61^d^	8.87 ± 2.87^c^	1.991	<0.001	<0.001	<0.001
EBI	276.11 ± 15.05^c^	293.60 ± 16.53^bc^	341.67 ± 35.88^a^	336.00 ± 11.28^a^	317.54 ± 15.37^ab^	8.055	0.014	0.008	0.019

BW, birth weight; ADG, average daily gain; ADFI, average daily feed intake; F/G; feed to gain ratio; mortality, the number of deaths within chicks and within a particular period of time; EBI, European Broiler Index. Values are presented as the mean ± SD. Means ± SD with unlike superscript letters in the same row are significantly different (*p* < 0.05). The same letter of the shoulder tag was considered to be not significant.

### 3.2 Organ index

As shown in Table 4, at 21 days of age, the cardiac index of broilers was significantly increased (*p* < 0.05) in groups supplemented with 0.5 and 1% CHM-JM113. The liver index of 0.25, 0.5 and 2% CHM-JM113 was significantly higher than that of control group (*p* < 0.01), and the addition of 0.5% CHM-JM113 was found to be the most effective. The spleen index of the 0.5 and 1% CHM-JM113 groups was significantly higher than that of the control group (*p* < 0.01). The broilers fed with diet containing 1 and 2% CHM-JM113 also had a higher bursa of Fabricius index than those fed with basal diet (*p* < 0.05). Dietary supplementation with CHM-JM113 linearly increased the bursa index (*p* < 0.05) and quadratically increased the cardiac index, liver index, and spleen index of broilers at 21 days of age (*p* < 0.05). At 42 days of age, the cardiac index of 1% CHM-JM113 was significantly higher than that of the 0.5% CHM-JM113 group (*p* < 0.05). The 0.5% supplementation level of CHM-JM113 could significantly (*p* < 0.01) decrease the liver index of broilers, while 0.25 and 2% CHM-JM113 could also significantly (*p* < 0.05) increase the spleen index of broilers. The dietary addition of CHM-JM113 linearly increased the spleen index and quadratically increased the liver index of 42-day-old broilers.

**Table 4:** Effect of experimental treatments on organ index of broilers (g/kg).

Item	Control	Dietary CHM-JM113 level (%)				SEM	*p*-value		
		0.25	0.5	1	2		ANOVA	Linear	Quadratic
**21 days**									
Cardiac	5.07 ± 0.77^c^	5.18 ± 0.93^bc^	5.97 ± 0.42^ab^	6.07 ± 0.61^a^	5.25 ± 0.32^bc^	0.135	0.030	0.146	0.017
Liver	20.97 ± 1.92^d^	26.55 ± 1.70^b^	32.59 ± 1.08^a^	23.51 ± 3.91^cd^	25.39 ± 2.37^bc^	0.828	<0.001	0.072	<0.001
Spleen	0.79 ± 0.32^b^	0.58 ± 0.10^b^	1.60 ± 0.60^a^	1.42 ± 0.31^a^	0.69 ± 0.18^b^	0.096	<0.001	0.161	<0.001
Bursa of fabricius	0.94 ± 0.18^c^	1.14 ± 0.10^c^	1.26 ± 0.14^bc^	1.58 ± 0.41^ab^	1.82 ± 0.54^a^	0.080	<0.001	<0.001	0.587
**42 days**									
Cardiac	5.21 ± 1.37^ab^	5.12 ± 0.51^ab^	4.39 ± 0.45^b^	6.37 ± 0.96^a^	5.39 ± 1.29^ab^	0.206	0.034	0.218	0.547
Liver	26.19 ± 1.87^a^	26.02 ± 3.51^a^	19.20 ± 2.94^b^	23.41 ± 3.25^a^	25.87 ± 2.27^a^	0.690	0.001	0.382	0.001
Spleen	0.68 ± 0.11^b^	0.92 ± 0.20^a^	0.91 ± 0.09^ab^	0.80 ± 0.19^ab^	1.02 ± 0.26^a^	0.038	0.035	0.025	0.647
Bursa of fabricius	0.31 ± 0.13	0.47 ± 0.18	0.28 ± 0.04	0.35 ± 0.11	0.43 ± 0.10	0.024	0.060	0.451	0.578

Values are presented as the mean ± SD (*n* = 6). Means ± SD with unlike superscript letters in the same row are significantly different (*p* < 0.05). The same letter of the shoulder tag was considered to be not significant.

### 3.3 Histomorphology of small intestine

**Figure 1:**
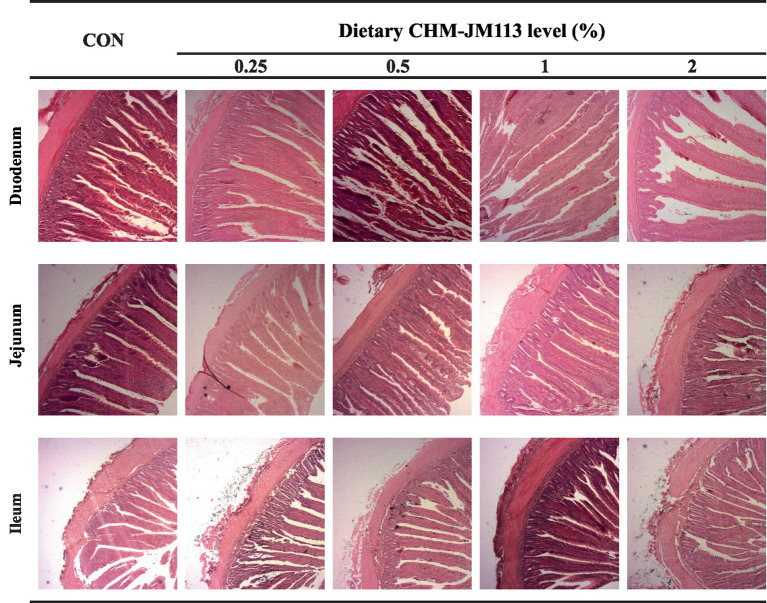
The duodenum, jejunum and ileum histological morphology of 21 days of broilers (hematoxylin and eosin). CON, broilers were fed with a basal diet; 0.25, 0.5, 1 and 2, broilers were fed with a basal diet supplemented with 0.25, 0.5, 1 and 2% CHM-JM113, respectively. Scale bars = 200 μm.

**Figure 2:**
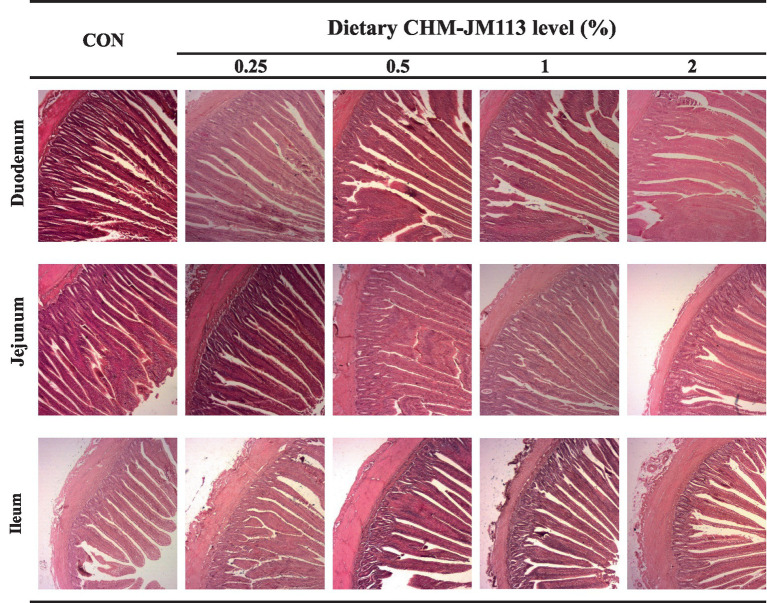
The duodenum, jejunum and ileum histological morphology of 42 days of broilers (hematoxylin and eosin). CON, broilers were fed with a basal diet; 0.25, 0.5, 1 and 2, broilers were fed with a basal diet supplemented with 0.25, 0.5, 1 and 2% CHM-JM113, respectively. Scale bars = 200 μm.

The light microscope images of the intestinal morphology are shown in Figures 1, 2. The effects of CHM-JM113 on the intestinal morphology of broilers are shown on Tables 5, 6.

**Table 5:** Effects of different experimental treatments on intestinal morphology of 21-day-old broilers.

Items	Control	Dietary CHM-JM113 level (%)				SEM	*p*-value		
		0.25	0.5	1	2		ANOVA	Linear	Quadratic
**Duodenum**									
VH, μm	1374.93 ± 5.38^b^	1376.44 ± 9.17^b^	1385.04 ± 3.49^ab^	1388.57 ± 10.92^ab^	1398.16 ± 4.71^a^	2.771	0.017	0.001	0.498
CD, μm	223.56 ± 2.58	223.42 ± 1.60	222.25 ± 2.62	222.15 ± 1.26	221.34 ± 3.26	0.565	0.760	0.217	0.961
VH/CD	6.15 ± 0.06^b^	6.16 ± 0.03^b^	6.23 ± 0.06^ab^	6.25 ± 0.07^ab^	6.32 ± 0.09^a^	0.022	0.045	0.004	0.658
**Jejunum**									
VH, μm	891.46 ± 7.19	894.32 ± 1.47	903.72 ± 9.92	907.91 ± 0.90	906.83 ± 11.59	2.438	0.076	0.009	0.443
CD, μm	192.06 ± 1.45	190.04 ± 1.88	191.68 ± 1.74	192.27 ± 1.57	188.85 ± 1.90	0.514	0.139	0.211	0.326
VH/CD	4.64 ± 0.006^b^	4.70 ± 0.05^b^	4.72 ± 0.03^b^	4.72 ± 0.04^b^	4.80 ± 0.07^a^	0.016	0.013	0.001	0.722
**Ileum**									
VH, μm	753.64 ± 2.05^c^	764.37 ± 2.03^c^	783.81 ± 4.05^b^	817.77 ± 17.51^a^	796.03 ± 7.92^b^	6.382	<0.001	<0.001	0.025
CD, μm	146.54 ± 0.64	141.61 ± 3.56	144.29 ± 3.67	142.66 ± 3.46	144.52 ± 1.79	0.775	0.337	0.582	0.168
VH/CD	5.14 ± 0.01^c^	5.40 ± 0.13^bc^	5.44 ± 0.14^b^	5.74 ± 0.27^a^	5.51 ± 0.04^ab^	0.060	0.008	0.003	0.048

VH, villus height; CD, crypt depth; VH/CD, villus height/crypt depth. Values are presented as the mean ± SD (*n* = 6). Means ± SD with unlike superscript letters in the same row are significantly different (*p* < 0.05). The same letter of the shoulder tag was considered to be not significant.

**Table 6:** Effects of different experimental treatments on intestinal morphology of 42-day-old broilers.

Items	Control I	Levels of CHM-JM113 added to diets (%)				SEM	*p*-value		
		0.25	0.5	1	2		ANOVA	Linear	Quadratic
**Duodenum**									
VH, μm	1473.05 ± 1.10^b^	1480.20 ± 6.20^b^	1508.29 ± 24.22^ab^	1519.94 ± 32.01^a^	1520.01 ± 15.50^a^	6.809	0.036	0.004	0.483
CD, μm	215.74 ± 2.81	215.21 ± 2.37	214.92 ± 0.67	214.78 ± 0.48	214.70 ± 0.81	0.390	0.944	0.443	0.788
VH/CD	6.83 ± 0.10^b^	6.88 ± 0.09^ab^	7.02 ± 0.11^ab^	7.08 ± 0.14^a^	7.08 ± 0.08^a^	0.036	0.041	0.004	0.463
**Jejunum**									
VH, μm	1032.20 ± 15.82^b^	1040.63 ± 18.24^b^	1073.76 ± 18.29^a^	1076.62 ± 17.24^a^	1074.66 ± 15.18^a^	6.314	0.021	0.003	0.195
CD, μm	207.87 ± 1.48	205.12 ± 1.55	205.25 ± 1.48	204.32 ± 2.29	205.40 ± 1.83	0.499	0.214	0.103	0.112
VH/CD	4.97 ± 0.04^c^	5.07 ± 0.11^bc^	5.23 ± 0.05^ab^	5.27 ± 0.14^a^	5.23 ± 0.04^ab^	0.037	0.007	0.001	0.056
**Ileum**									
VH, μm	789.86 ± 4.17^d^	812.89 ± 4.82^c^	829.75 ± 12.66^b^	855.74 ± 7.07^a^	843.74 ± 8.65^ab^	6.444	<0.001	<0.001	0.006
CD, μm	146.46 ± 3.06^bc^	144.81 ± 3.11^b^	147.09 ± 1.32^bc^	150.22 ± 0.88^ab^	151.25 ± 0.21^a^	0.786	0.018	0.003	0.196
VH/CD	5.39 ± 0.11^b^	5.61 ± 0.13^a^	5.64 ± 0.14^a^	5.70 ± 0.02^a^	5.58 ± 0.06^a^	0.035	0.037	0.033	0.014

VH, villus height; CD, crypt depth; VH/CD, villus height/crypt depth. Values are presented as the mean ± SD (*n* = 6). Means ± SD with unlike superscript letters in the same row are significantly different (*p* < 0.05). The same letter of the shoulder tag was considered to be not significant.

Table 5 demonstrates that in 21-day-old broilers, 2% CHM-JM113 resulted in increased VH and VH/CD ratio in the duodenum, as well as an increased VH/CD ratio in the jejunum (*p* < 0.05). These indices were also linearly elevated in comparison to the control group (*p* < 0.01). VH and VH/CD ratio in the ileum were significantly, linearly and quadratically enhanced (*p* < 0.01) when 0.5, 1, and 2% CHM-JM113 were added to the basal diet.

As observed in Table 6, at 42 days of age, 1 and 2% CHM-JM113 significantly (*p* < 0.05) increased VH and VH/CD ratio in the duodenum of broilers, while showing a linear effect (*p* < 0.01). The VH and VH/CD ratio in the jejunum of the 0.5, 1, and 2% CHM-JM113 groups was significantly higher than that of the control group. Additionally, the jejunal VH and VH/CD ratio values showed a linear increase with the addition of CHM-JM113 (*p* < 0.01). Meanwhile, the VH and VH/CD ratio of the ileum in CHM-JM113 treated groups with different levels of supplementation were significantly greater than those of the control group, and the addition of CHM-JM113 enhanced these indices in both quadratically and linearly (*p* < 0.05). Notably, the addition of 2% CHM-JM113 treatment group significantly and linearly increased CD values in the ileum of broilers (*p* < 0.05).

### 3.4 Serum antioxidant capacity

The impact of CHM-JM113 on the antioxidant properties of broilers is presented in Table 7. Compared with the control group, at 21 days of age, 0.5 and 1% CHM-JM113 significantly increased the serum SOD concentration of broilers (*p* < 0.01). Conversely, the addition of different levels of CHM-JM113 highly significantly decreased the serum MDA concentration (*p* < 0.01), and this reduction effect was dose-dependent. At 42 days of age, the SOD concentrations of 0.5, 1 and 2% CHM-JM113 were significantly higher than those of the control group (*p* < 0.01). The highest SOD concentration was found in 1% CHM-JM113. Additionally, the MDA concentrations in broiler serum were significantly reduced by different levels of CHM-JM113 (*p* < 0.01), and the best reduction observed in 0.5% CHM-JM113. The inclusion of 1 and 2% levels of CHM-JM113 notably increased the T-AOC level of broilers (*p* < 0.05). Furthermore, 2% CHM-JM113 significantly reduced the CAT level of broilers (*p* < 0.05).

**Table 7:** Effects of different experimental treatments on antioxidant properties of broiler.

Items	Control	Dietary CHM-JM113 level (%)				SEM	*p*-value		
		0.25	0.5	1	2		ANOVA	Linear	Quadratic
**21 days**									
SOD, U/mL	32.71 ± 0.85^b^	38.4 ± 8.90^b^	51.45 ± 3.49^c^	90.23 ± 6.21^d^	15.68 ± 0.66^a^	6.789	<0.001	0.088	<0.001
MDA, nmol/mL	1.75 ± 0.01^e^	1.62 ± 0.03^d^	1.46 ± 0.02^c^	1.18 ± 0.01^b^	0.83 ± 0.01^a^	0.088	<0.001	<0.001	<0.001
T-AOC, U/mL	0.64 ± 0.07	0.67 ± 0.03	0.76 ± 0.06	0.85 ± 0.03	0.87 ± 0.21	0.034	0.065	0.006	0.930
CAT, U/mL	10.51 ± 1.56	7.36 ± 1.29	10.38 ± 1.43	8.40 ± 1.09	8.84 ± 2.07	0.462	0.126	0.427	0.522
**42 days**									
SOD, U/mL	39.09 ± 3.50^a^	44.87 ± 2.84^a^	63.18 ± 0.99^b^	151.37 ± 1.26^c^	63.75 ± 6.74^b^	10.895	<0.001	<0.001	<0.001
MDA, nmol/mL	1.66 ± 0.02^d^	1.62 ± 0.01^c^	1.41 ± 0.01^a^	1.42 ± 0.03^a^	1.52 ± 0.02^b^	0.027	<0.001	<0.001	<0.001
T-AOC, U/mL	0.68 ± 0.03^a^	0.71 ± 0.01^ab^	0.73 ± 0.01^abc^	0.77 ± 0.02^bc^	0.80 ± 0.08^bc^	0.015	0.029	0.002	0.826
CAT, U/mL	15.18 ± 2.52^b^	15.30 ± 0.21^b^	16.96 ± 1.22^b^	16.46 ± 1.41^b^	10.07 ± 3.06^a^	0.783	0.011	0.030	0.005

Values are presented as the mean ± SD (*n* = 6). Means ± SD with unlike superscript letters in the same row are significantly different (*p* < 0.05). The same letter of the shoulder tag was considered to be not significant.

### 3.5 Gene expression

**Figure 3:**
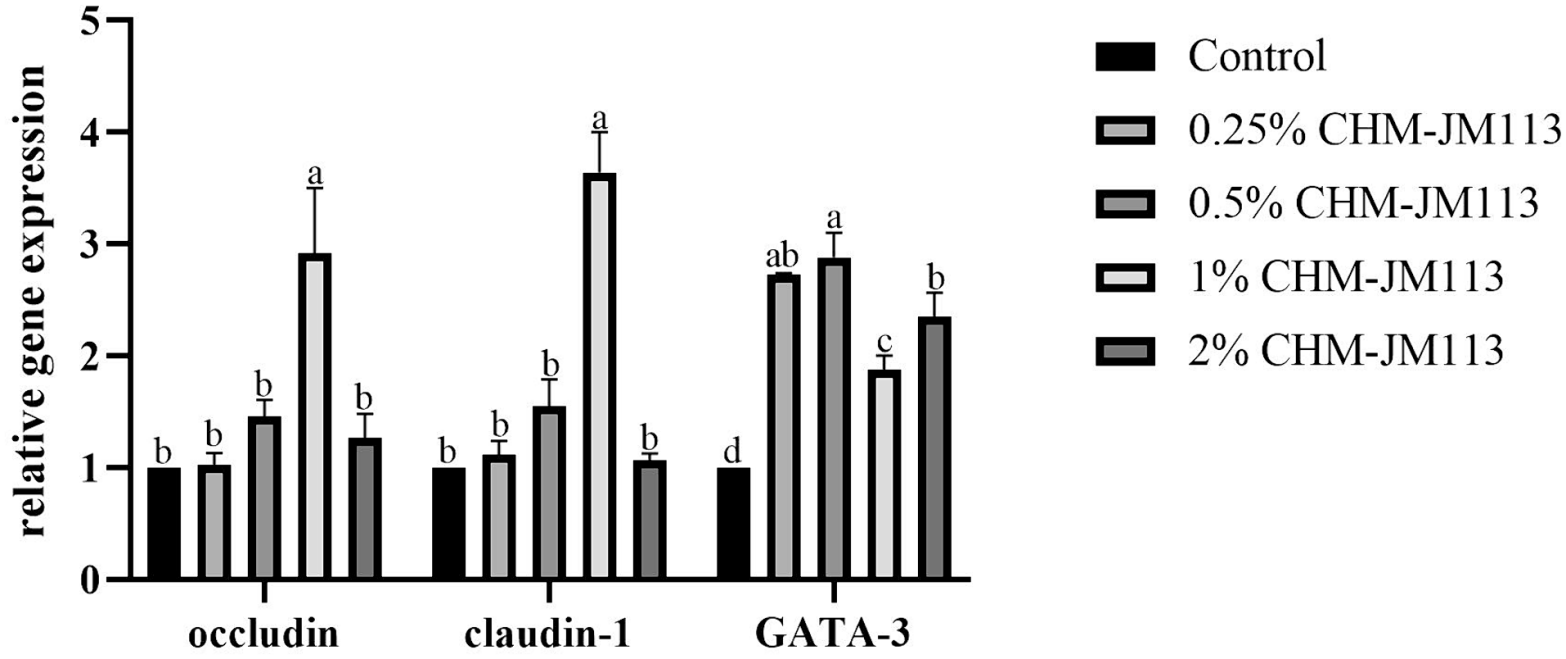
Effects of CHM-JM113 on gene expression levels of occludin, claudin-1 and GATA-3 in duodenum of broilers (day 21).

As shown in Figure 3, adding 1% of CHM-JM113 to the duodenum of broiler chickens was found to significantly increase the level of occludin (*p* < 0.05) and to very significantly increase the levels of claudin-1 and GATA-3 (*p* < 0.01). Adding varying amounts of CHM-JM113 was found to promote the expression of GATA-3 in the duodenum (*p* < 0.01), with the 0.5% addition having the greatest effect in the duodenum (*p* < 0.01).

**Figure 4:**
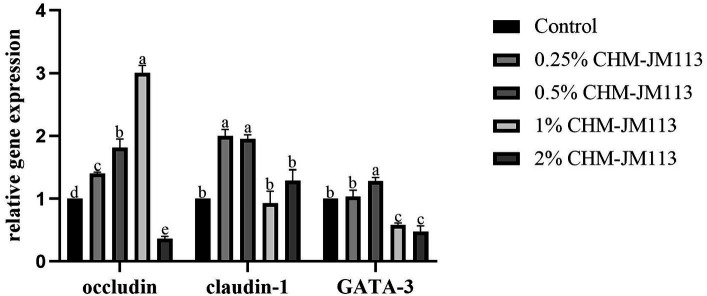
Effects of CHM-JM113 on gene expression levels of occludin, claudin-1 and GATA-3 in duodenum of broilers (day 42).

As can be seen from Figure 4, the addition of 0.25, 0.5 and 1% CHM-JM113 all significantly increased the expression level of occludin in the duodenum of broiler chickens extremely significantly (*p* < 0.01). The promotion effect increased linearly, but the intestinal occludin expression level of 2% addition level of CHM-JM113 was significantly lower than that of the control group (*p* < 0.01). The claudin-1 expression level of CHM-JM113 added at 0.25 and 0.5% was significantly higher than that of the control group (*p* < 0.01), and there was no significant difference in the promotional effect of the two addition levels of CHM-JM113 on claudin-1 expression in the jejunum (*p* > 0.05). The addition of 0.5% CHM-JM13 significantly increased the 42-day-old GATA-3 levels in broilers (*p* < 0.01), but the addition of 1 and 2% CHM-JM113 instead decreased GATA-3 levels (*p* < 0.01).

**Figure 5:**
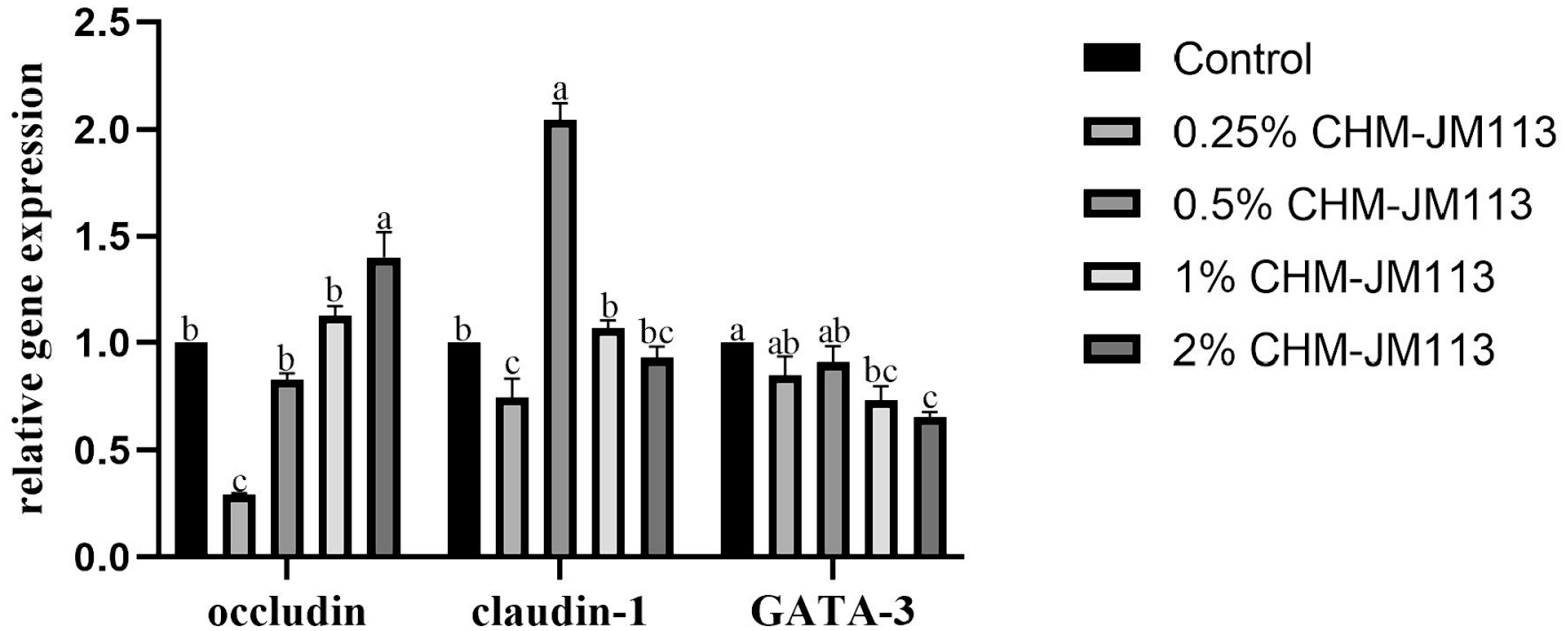
Effects of CHM-JM113 on gene expression levels of occludin, claudin-1 and GATA-3 in jejunum of broilers (day 21).

As can be seen from Figure 5, the adding of different proportions of CHM-JM113 had a linear effect on the expression of occludin in the intestines (*p* < 0.01). However, only at the addition of 2% CHM-JM113, the expression level was significantly higher than that of the control group (*p* < 0.01). The addition of 0.25% CHM-JM113 extremely significantly decreased the expression level of claudin-1 in the jejunum of 21-day-old broilers (*p* < 0.01). On the other hand, 0.5% CHM-JM113 significantly increased the expression of claudin-1 (*p* < 0.01), and 1 and 2% CHM-JM113 significantly decreased the level of GATA-3 expression in the jejunum (*p* < 0.05).

**Figure 6:**
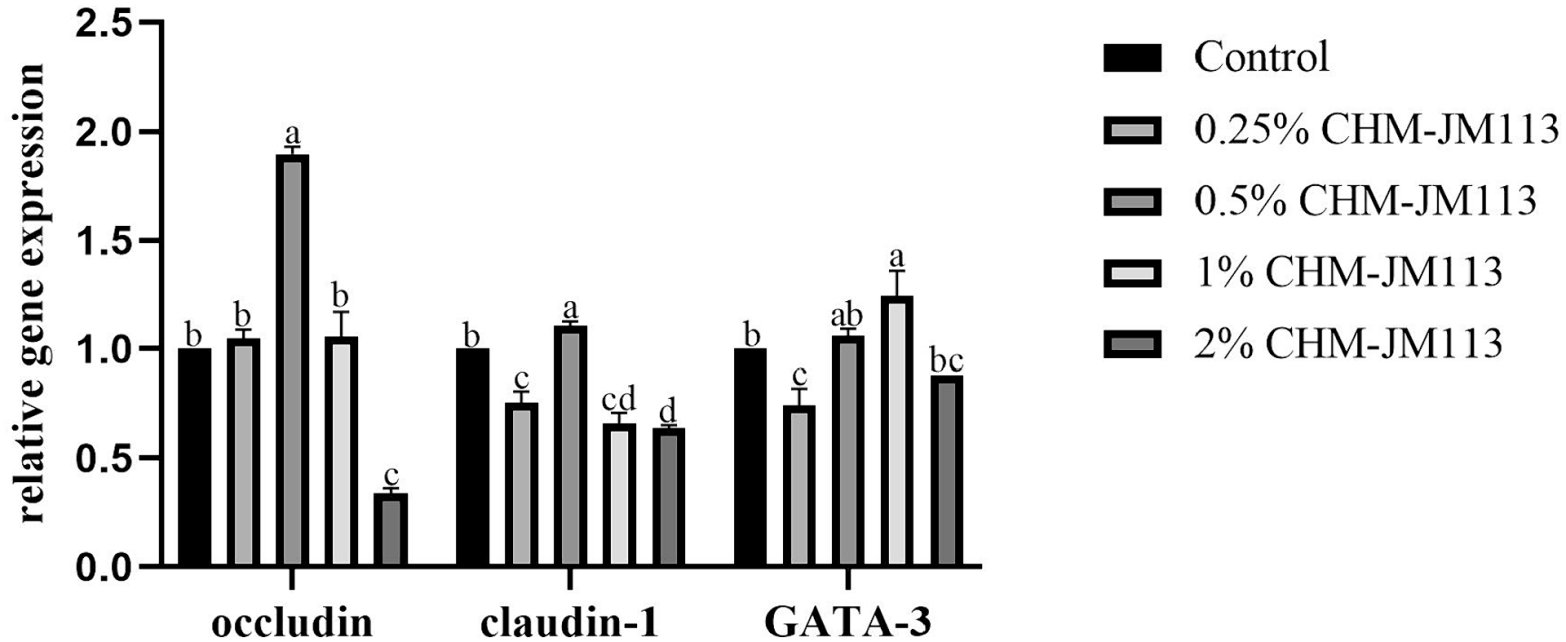
Effects of CHM-JM113 on gene expression levels of occludin, claudin-1 and GATA-3 in jejunum of broilers (day 42).

As can be seen in Figure 6, 0.5% CHM-JM113 significantly increased the occludin expression level in 42-day-old broilers (*p* < 0.01), while 2% CHM-JM113 significantly decreased its expression level (*p* < 0.01). Additionally, 0.5% CHM-JM113 significantly increased the claudin-1 expression level (*p* < 0.01); however, other levels of CHM-JM113 significantly decreased the expression of claudin-1 (*p* < 0.01). Furthermore, 0.25% CHM-JM113 significantly decreased the expression of GATA-3 in the jejunum of broilers (*p* < 0.01), and 1% CHM-JM113 highly significantly promoted the expression of GATA-3 (*p* < 0.01).

## 4 Discussion

### 4.1 Growth performance

Chinese herbal medicine is able to promote the proliferation of probiotics and has a turnover effect on intestinal flora imbalance. Probiotics also facilitate the dissolution of active ingredients of Chinese herbal medicine, reduce the toxic and side effects of Chinese herbal medicine (30), and improve its efficacy. Wang et al. (31) showed that *Lactobacillus plantarum* JM113 has the ability to inhibit the growth of Salmonella in broilers, reduce the incidence of broiler chickens, and reduce the mortality. Liang et al. (32) found that the combination of Chinese herbal medicine and lactic acid bacteria could enhance the intestinal sensitivity of broilers, inhibit the growth of *Escherichia coli*, and reduce the diarrhea rate and mortality of broilers. Consistent with the results of this experiment, the addition of CHM-JM113 in the diet significantly reduced the mortality of broilers. Both herbs and probiotics have been shown to promote growth in animals (33, 34). Theoretically, the growth-promoting effects of herbs and probiotics in broilers should have a cumulative effect, but in fact, in this study, the compound herbs and JM113 did not have a significant growth-promoting effect on broilers (*p* > 0.05), which was similar to the results of Majekodunmi et al. (35), who found that there was no significant difference in feed intake and weight gain of Ross broilers with sweet citrus peel. Sun et al. (36) found that dietary supplementation with *Bacillus amyloliquefaciens* CECT 5940 did not significantly promote the growth of broilers fed a corn-soybean meal diet. This may be due to the antibacterial effect of compound Chinese herbal medicine and probiotics (37), rather than their growth-promoting effects. Several studies have demonstrated that thyme, Astragalus, tangerine peel, and dandelion have antioxidant and immune-promoting effects (38–40) and partially growth-promoting effects (24). Furthermore, the intricate and variability of the synergistic potential of plant extract combinations (41) may be a contributing factor to the lack of improvement in broiler growth performance in this experiment. The strain specificity of the host’s immune and gut health response to probiotics (42) may also be a contributing factor to the non-significant differences in growth performance in this experiment.

### 4.2 Organ index

To a certain extent, organ index could reflect the development of animal organs, body weight changes and indirectly reflect the ability of animals to cope with external stress. The higher the index, the stronger the body’s immunity (43). The bursa of Fabricius is the site where B lymphocytes mature, and the spleen regulates the cellular immunity and humoral immunity of poultry by activating T lymphocytes, B lymphocytes and macrophages (44). Liang et al. (32) found that the combination of Chinese herbal medicine and probiotics improved the spleen index, thymus index and bursa index of broilers. This combined treatment had a more significant effect compared to using Chinese herbal medicine or probiotics alone. Li et al. (45) found that Astragalus polysaccharides significantly improved the thymus index and spleen index *in vivo* experiments, and were useful in restoring the damaged thymus and spleen tissue structure. In this experiment, the enhancement of the immune organ index in broilers in different degrees verified the previous perspective. The liver is the largest solid gland and an important metabolic organ, plays a vital role in excretion, metabolism, detoxification and the production of various coagulation factors (46). The growth performance of broilers may also decrease to a certain extent after liver damage (47). This experiment found that adding 0.3 g/mL of a compound Chinese herbal medicine with JM113 improved the liver index of broilers, indicating that Chinese herbal medicine is beneficial to liver health, which is consistent with previous studies (48). It is known that the combined use of compound herbs and JM113 promotes the development of the intestinal tract, thereby boosting the absorption of nutrients by the animal organism, which in turn supports the improvement of the organ index of broilers. The addition of CHM-JM113 in this experiment boosted the development of broiler organ indices to varying degrees, but was significantly influenced by the dosage, suggesting that the use of CHM-JM113 had a positive impact on the immunological performance of broilers.

### 4.3 Histomorphology of small intestine

The integrity of intestinal structure is very important for animals to absorb nutrients. Intestinal villus height (VH), crypt depth (CD) and VH/CD ratio are important indicators of intestinal structural integrity (49). Longer intestinal villus height and shallower crypt depth indicate better healthy digestion and absorption of microorganisms (10), improved intestinal mucosal differentiation, and enhanced capacity for digestion and absorption (50). Previous studies have found that adding probiotics, forsythia extract, natural pepper extract, and ellagic acid is an alternative to antibiotics in increasing the intestinal villus height of broilers (51, 52). This experiment found that compared with the control group, the combination of compound Chinese herbal medicine and JM113 improved the villus height and villus height ratio of the duodenum, jejunum and ileum of broilers, which is consistent with previous studies. This may be due to the fact that lactic acid bacteria, acting as probiotics, convert dietary fiber into short-chain fatty acids. They also promote the development of intestinal villi, improve the integrity of the intestinal barrier, and consequently improve the absorption of nutrients (53). Chinese herbal medicine is abundant in active ingredients, including lipids, unsaturated fatty acids, sugars, trace elements and vitamins, which provide sufficient nutrition for intestinal villus epithelial cells and support intestinal development (26). Studies have shown that there is a two-way communication between the liver and the intestine. Bile acids produced in the liver regulate the microbial composition and intestinal barrier function, while intestinal products regulate bile acid synthesis, as well as glucose and lipid metabolism in the liver. The intestinal barrier serves to restrict the systemic transmission of microorganisms and toxins, while facilitating the passage of nutrients into the portal vein circulation for delivery to the liver (54). Based on the gut-liver relationship, the increase of liver index is also benefits the absorption of nutrients by the intestine. In this experiment, the addition of compound Chinese herbal medicine and JM113 not only increased the liver index but also increased the absorption of nutrients in the small intestine, possibly as a result of the bidirectional communication between the liver and the intestine. The combined use of compound Chinese herbal medicine and JM113 can be observed to promote the development of the intestine, thereby enhancing the absorption of nutrients by the animal body and consequently improving the organ index of broilers.

### 4.4 Serum antioxidant capacity

Oxidative stress refers to the imbalance between antioxidants and free radicals, which may lead to the production of various reactive oxygen species (ROS) such as hydroxyl radicals and superoxide anions in the body (55). Excess ROS can disrupt animal metabolism, damage cell structure, and accelerate oxidation, leading to tissue damage and various diseases (56). Antioxidant enzyme activity and oxidation product concentration are reliable biomarkers for assessing the antioxidant status of animals (50). SOD catalyzes the disproportionation of superoxide anions into oxygen and H_2_O_2_ (57). CAT is a common enzyme that catalyzes the decomposition of H_2_O_2_ into water and oxygen. In addition, previous studies have shown that the removal rate of H_2_O_2_ removal in cells is partially dependent on GSH levels (58). The reduction reaction of lipid peroxide is catalyzed by glutathione peroxidase (GSH-Px), and the overall antioxidant capacity is indicated by the level of T-AOC (59). MDA is one of the end products of peroxidation derived from membrane lipids, reflecting the extent of lipid peroxidation and damage to antioxidant capacity in animals (60). Yin et al. (59) found that adding LBPs to the diet could increase the serum total antioxidant capacity (T-AOC) and GSH-Px activity, and decrease the MDA level (*p* < 0.05). Abo-Samaha et al. (61) found that adding licorice (0.4 g/L and 0.8 g/L) to drinking water resulted in antioxidant activity, leading to a reduction in MDA levels and an increase in GSH levels and CAT activity. Various active ingredients in thyme, Astragalus, tangerine peel and dandelion have been shown to reduce the effects of peroxides on animal bodies and enhance the body’s antioxidant capacity (62–64). In this experiment, the concurrent administration of Chinese herbal medicine and JM113 resulted in increased levels of SOD, T-AOC and MDA in 21-day-old broilers. Additionally, the SOD level of 42-day-old broilers was also improved. The liver is the primary site of free radical formation. Shati et al. (65) found that aqueous extracts of thyme and ginger have detoxifying and antioxidant effects on the liver and brain of mice. Antioxidants like thyme and rosemary have been utilized as therapeutic agents to alleviate liver damage (66). Additionally, dandelion root is also believed to have a beneficial effect on the gastrointestinal system by stimulating digestion and liver function (67). The increase in the liver index in this experiment also supports the positive impact of CHM-JM113 on the antioxidant performance of broilers from another perspective.

### 4.5 Gene expression

Nutrients entering the animal intestine will be selectively digested and absorbed by the intestine. This is due to the physical barrier on the intestinal surface, which prevents harmful substances from entering the bloodstream through the intestinal mucosa and reduces the damage caused by these substances to the body (68). The intestinal physical barrier is composed of tight junction structures (TJ) and mucosal epithelial cells. TJ regulate the permeability of the intestinal barrier by sealing the paracellular space between adjacent epithelial cells, thereby preventing the spread of microorganisms and antigens (69). This is also a key indicator for assessing intestinal health. The regulation of intestinal tight junctions is mediated by related proteins, such as occludin, claundin-1 and MUC2. Occludin and claudin-1 play an important role in maintaining epithelial cell polarity and regulating intestinal barrier permeability (70). The up-regulation of occludin may indicate an increase in cell permeability to nutrients, which facilitates the digestion and absorption of nutrients (71). GATA-3 is a significant regulator of T-cell differentiation and a Th2 cell-specific transcription factor (72). Gene-targeting studies have demonstrated that GATA-3 plays a vital role in the development and function of T cells, B cells, CD1 natural killer cells (NKT cells), natural killer cells (NK), and innate lymphoid cells (ILCs) (73). This experiment revealed that the addition of CHM-JM113 significantly enhanced the expression of occludin and claudin-1 in the intestine and to varying extents, improved the integrity of the intestinal barrier and organ indices. This may be due to the fact that the intestinal mucosal immune system influences the development of immune organs, including the spleen, thymus, and secondary immune organs, as well as immune responses (74), which in turn promotes the expression of occludin, claudin-1, and GATA-3 in the intestine, consistent with previous research findings (75).

In summary, CHM-JM113 may be considered as an alternative to antibiotics for broiler production, but it does not improve the performance of the animal and economic considerations need to be taken into account.

## 5 Conclusion

In summary, the use of CHM-JM113 has been shown to reduce the mortality of AA broilers, increase the European Broiler Index (EBI), and improve the organ index of broilers, particularly the immunological organ index. In addition, CHM-JM113 promoted the growth of villi in the small intestine, increased the VH/CD ratio of each intestinal segment, enhanced the organism’s antioxidant performance, and up-regulated the expression of occludin, claudin-1 and GATA-3 in the gut. CHM-JM113 can be used as a substitute for antibiotics, but the specific mechanism needs further study.

## Data availability statement

The original contributions presented in the study are included in the article/supplementary material, further inquiries can be directed to the corresponding author.

## Ethics statement

The animal study was approved by the Animal Welfare and Ethics Committee of Hebei University of Engineering. The study was conducted in accordance with the local legislation and institutional requirements.

## Author contributions

GF: Conceptualization, Investigation, Supervision, Writing – review & editing. MZ: Data curation, Formal analysis, Software, Writing – original draft. LW: Funding acquisition, Project administration, Writing – review & editing. YuaH: Investigation, Conceptualization, Methodology, Resources,Writing – original draft. RH: Validation, Resources, Writing–original draft. KQ: Investigation, Methodology, Writing–original draft. LY: Date curation, Visualization, Writing–review & editing. DZ: Resources, Methodology, Writing–original draft. YueH: Formal analysis, software, Writing–original draft. TM: Visualization, Vvalidation, Writing – original draft.

## Author note

Chinese herbal medicines used in this experiment, including thyme, Astragalus, tangerine peel and dandelion, do not violate the taboos of “eighteen antagonisms and nineteen antagonisms.” The results indicated that the compound Chinese herbal medicine selected in this experiment had no significant effect on the activity of JM113. The plate count showed that 1 g of JM113 powder contained 4.4 × 109 CFU of viable bacteria.
